# Supplementary figures and images for: Dynamic Translational Landscape Revealed by Genome-Wide Ribosome Profiling under Drought and Heat Stress in Potato
Source: Plants (Basel). 2023 Jun 6;12(12):2232. doi: 10.3390/plants12122232 (PMC10304021; doi:10.3390/plants12122232)

35°C 0 h

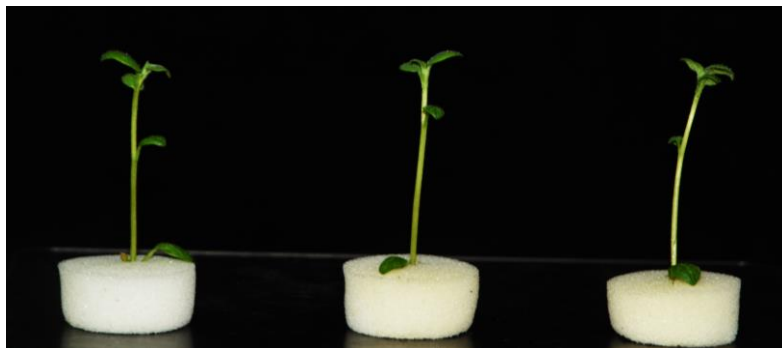

35°C 3 h

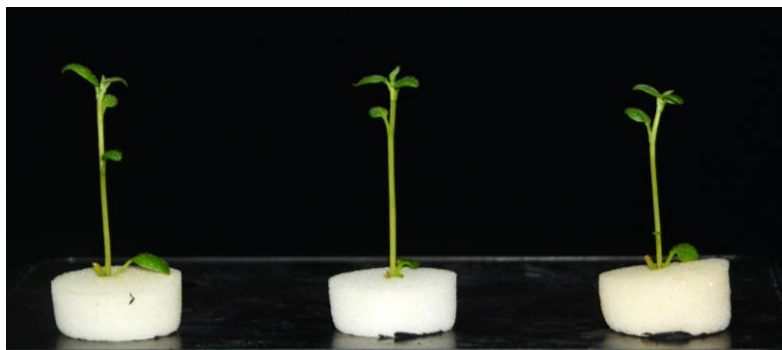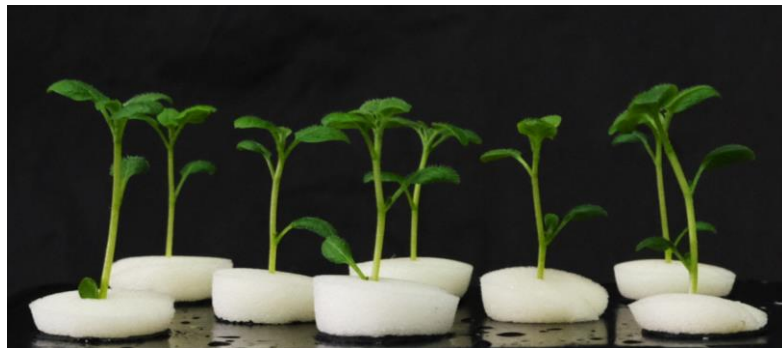

20%PEG6000 0 h

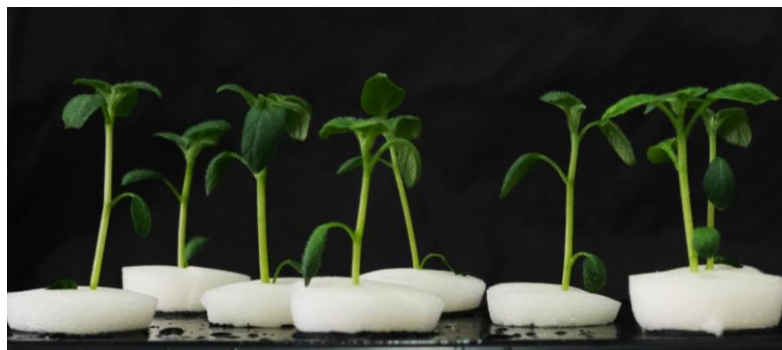

20%PEG6000 3 h

Supplement: Supplementary file 1 [file plants-12-02232-s001.zip › Supplementary files/Fig. S1.pdf]

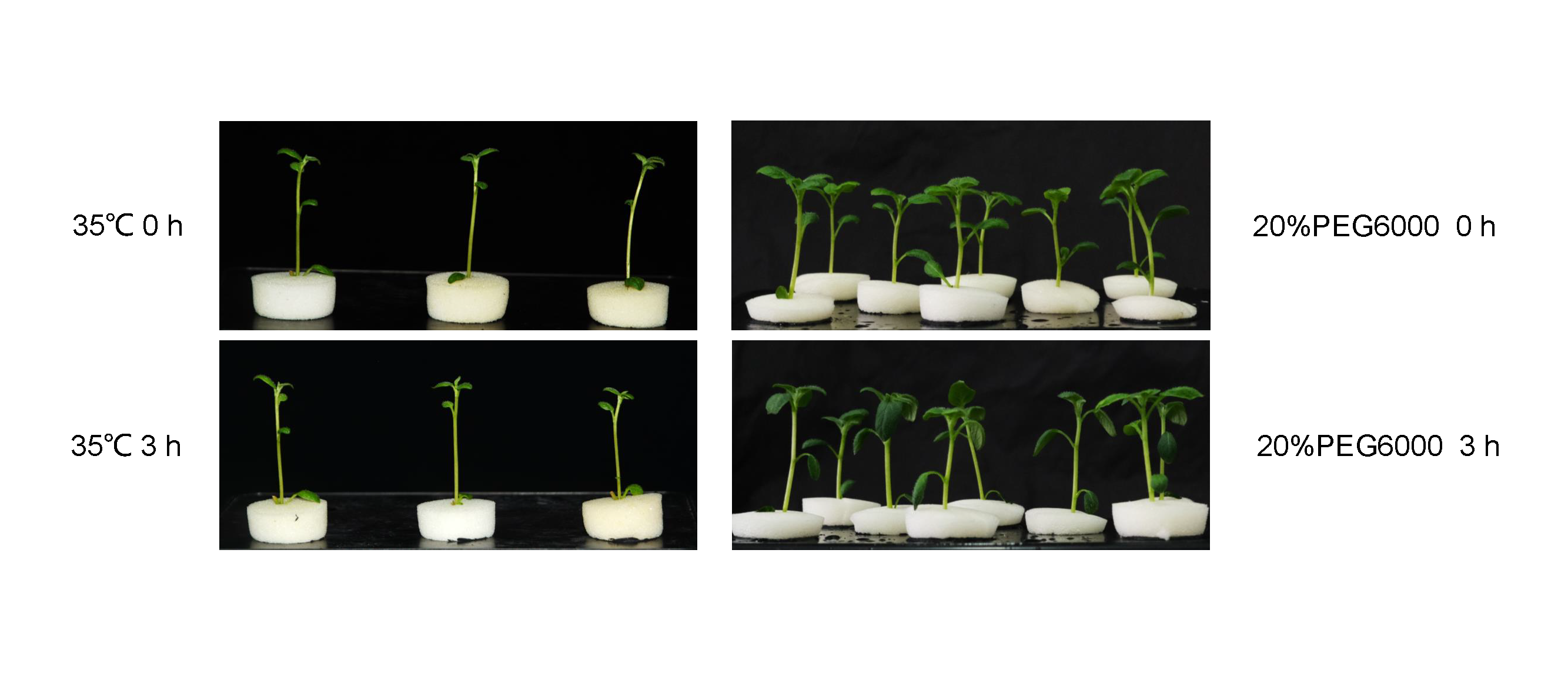

Supplement: Supplementary file 1 [file plants-12-02232-s001.zip › Supplementary files/Fig. S1.tif]

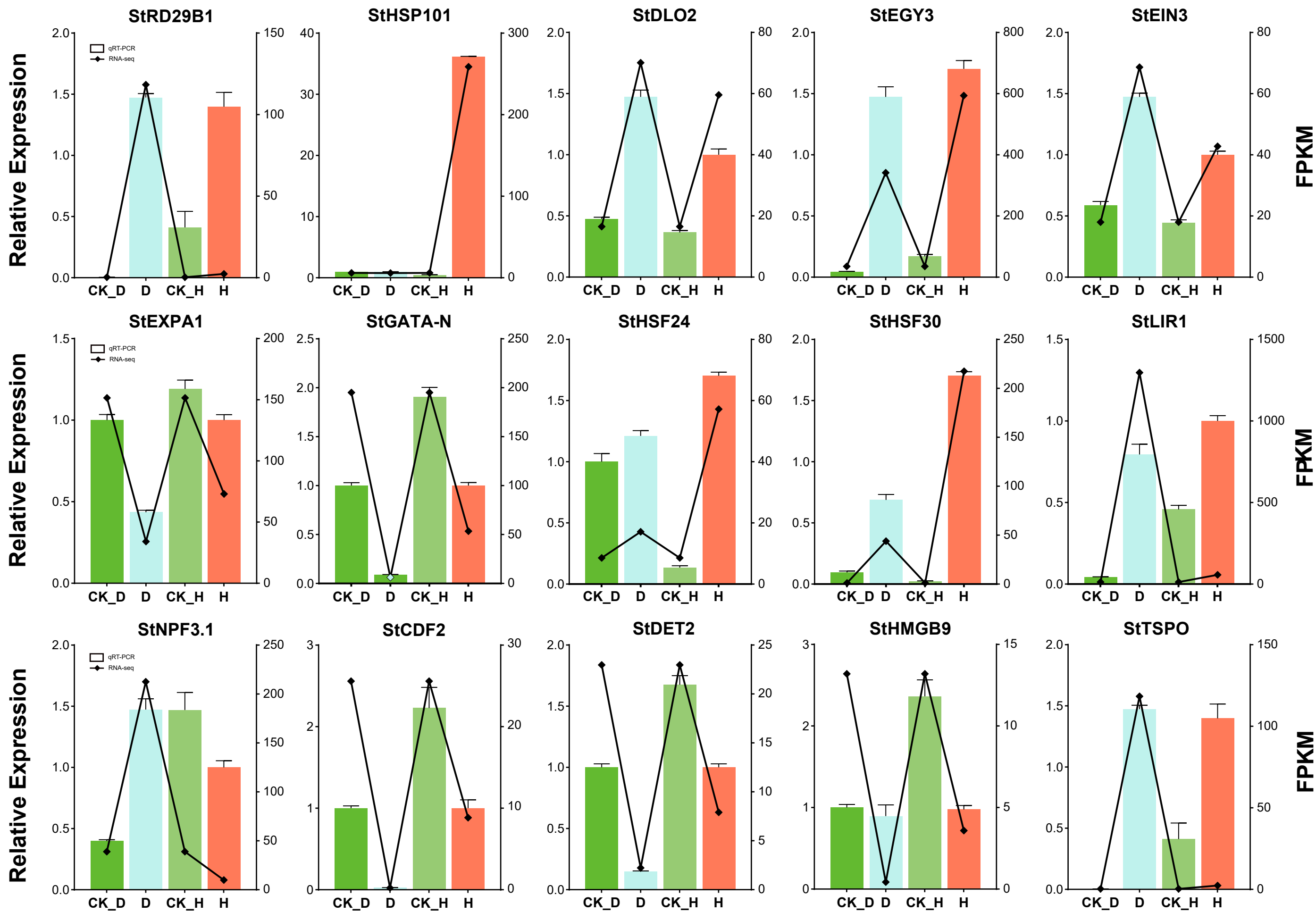

Supplement: Supplementary file 1 [file plants-12-02232-s001.zip › Supplementary files/Fig. S2.pdf]

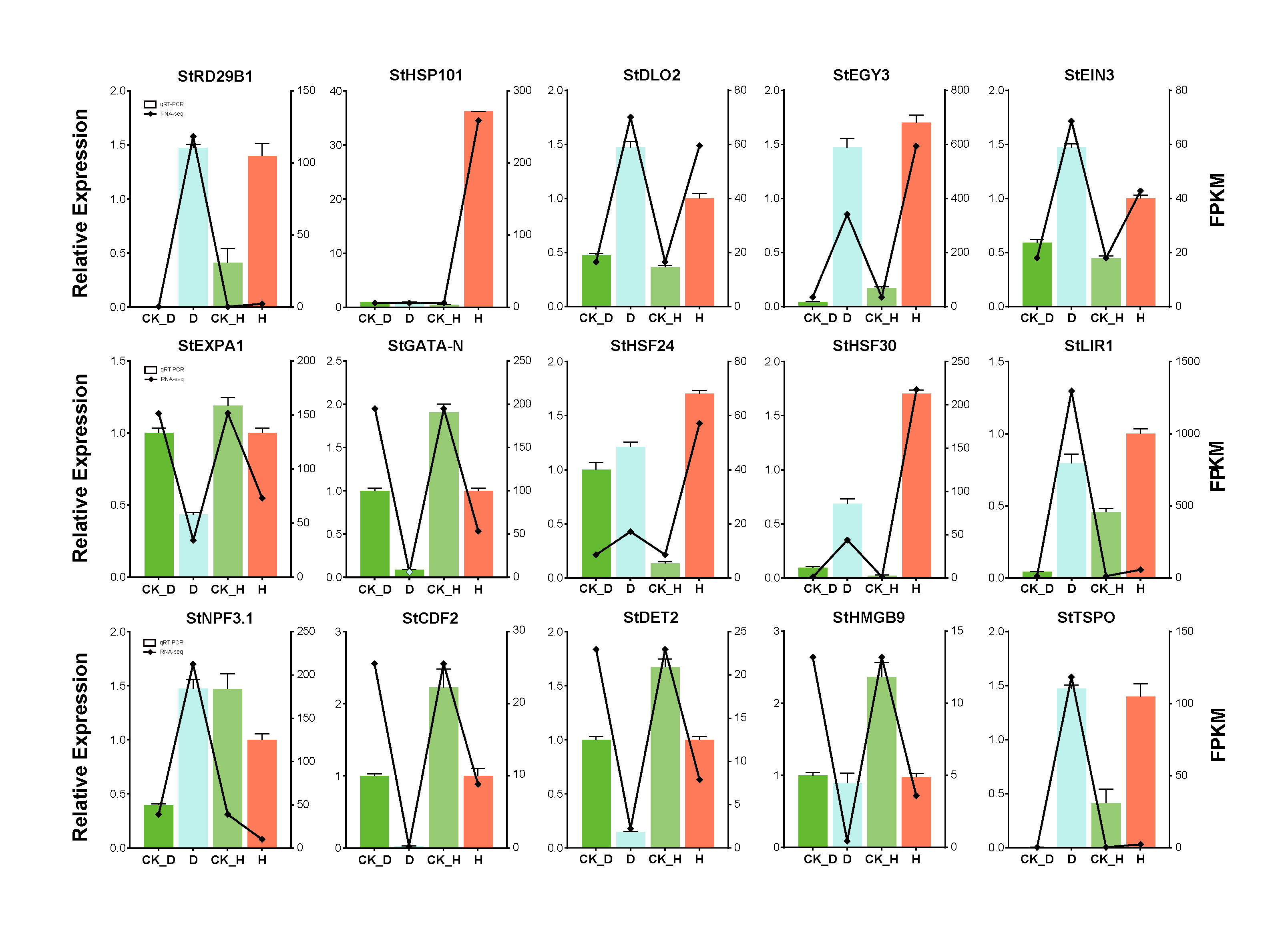

Supplement: Supplementary file 1 [file plants-12-02232-s001.zip › Supplementary files/Fig. S2.tif]

a)

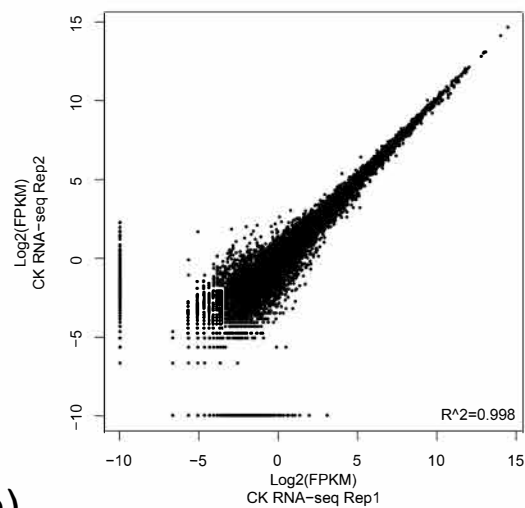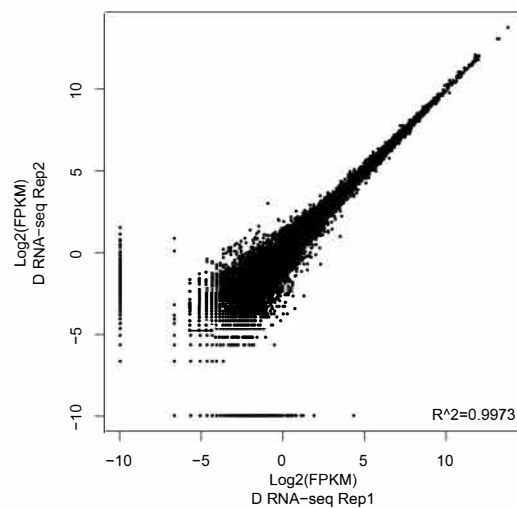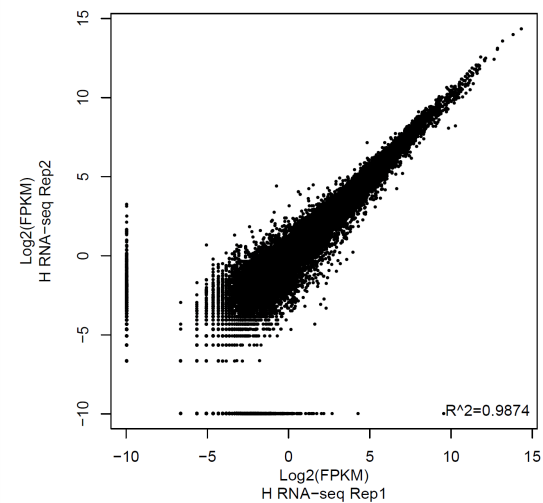

b)

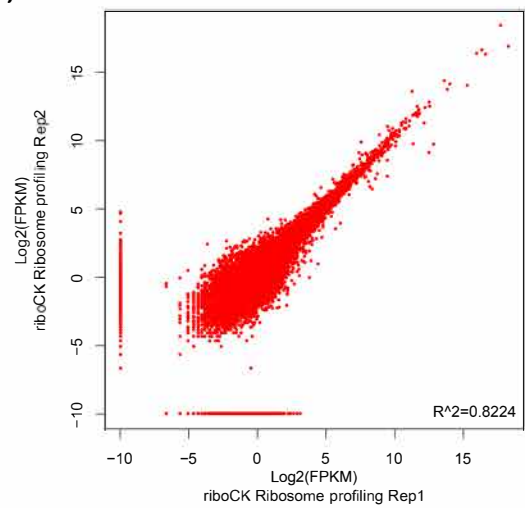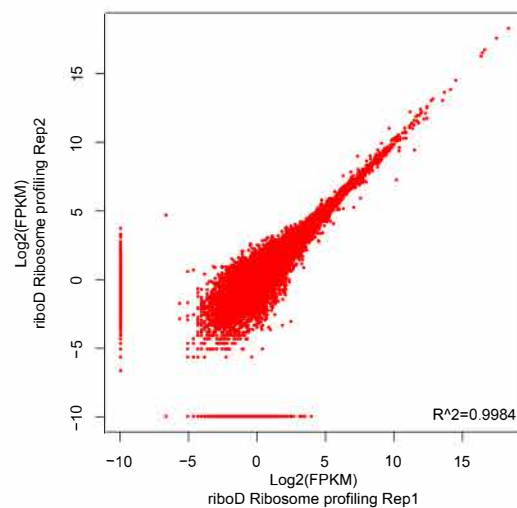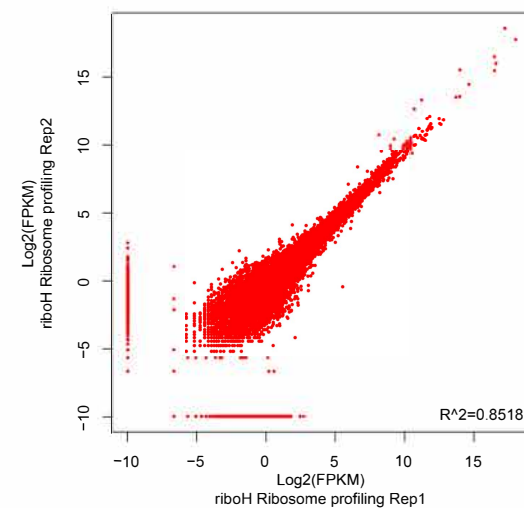

Supplement: Supplementary file 1 [file plants-12-02232-s001.zip › Supplementary files/Fig. S3.pdf]

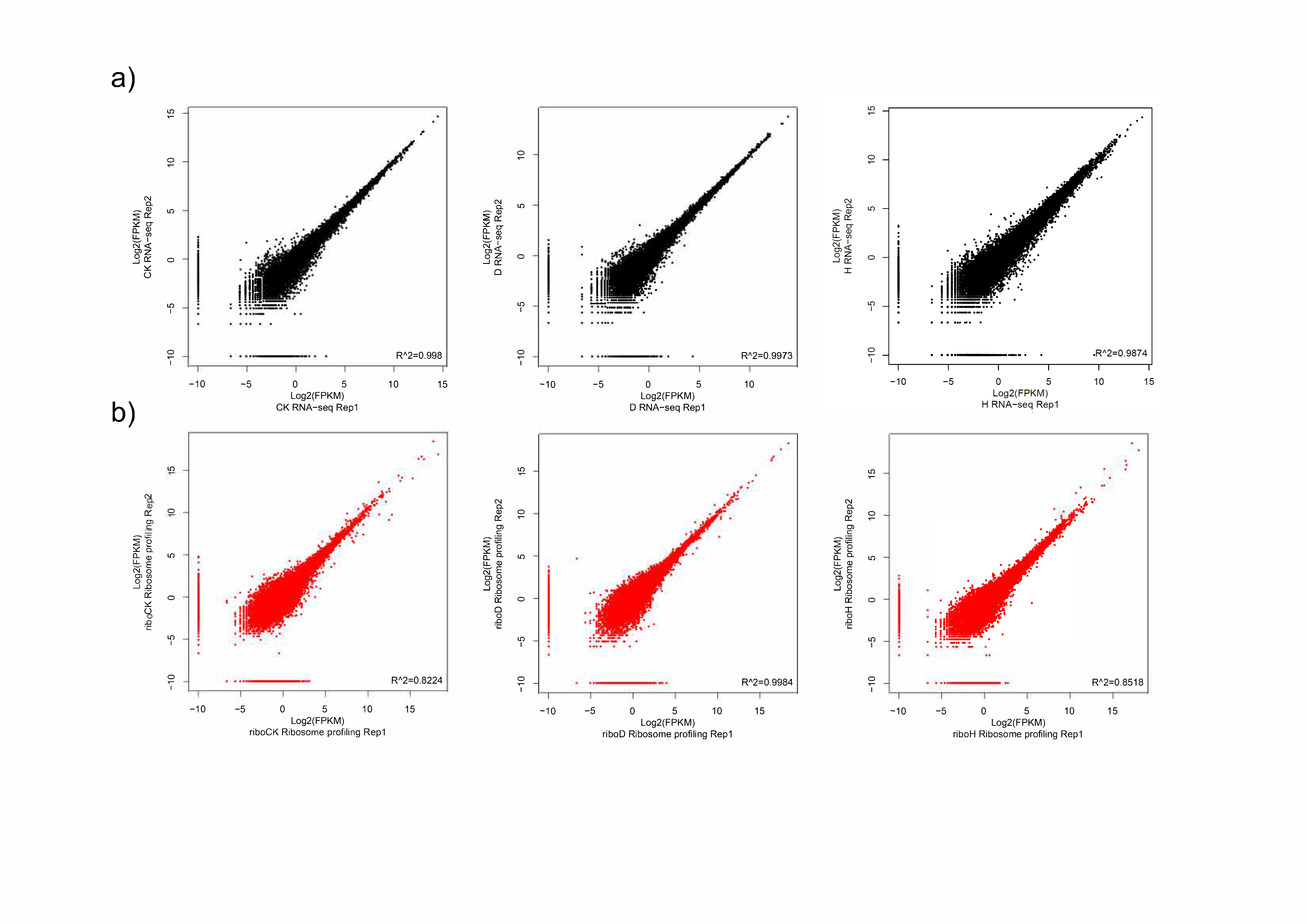

Supplement: Supplementary file 1 [file plants-12-02232-s001.zip › Supplementary files/Fig. S3.tif]

a)

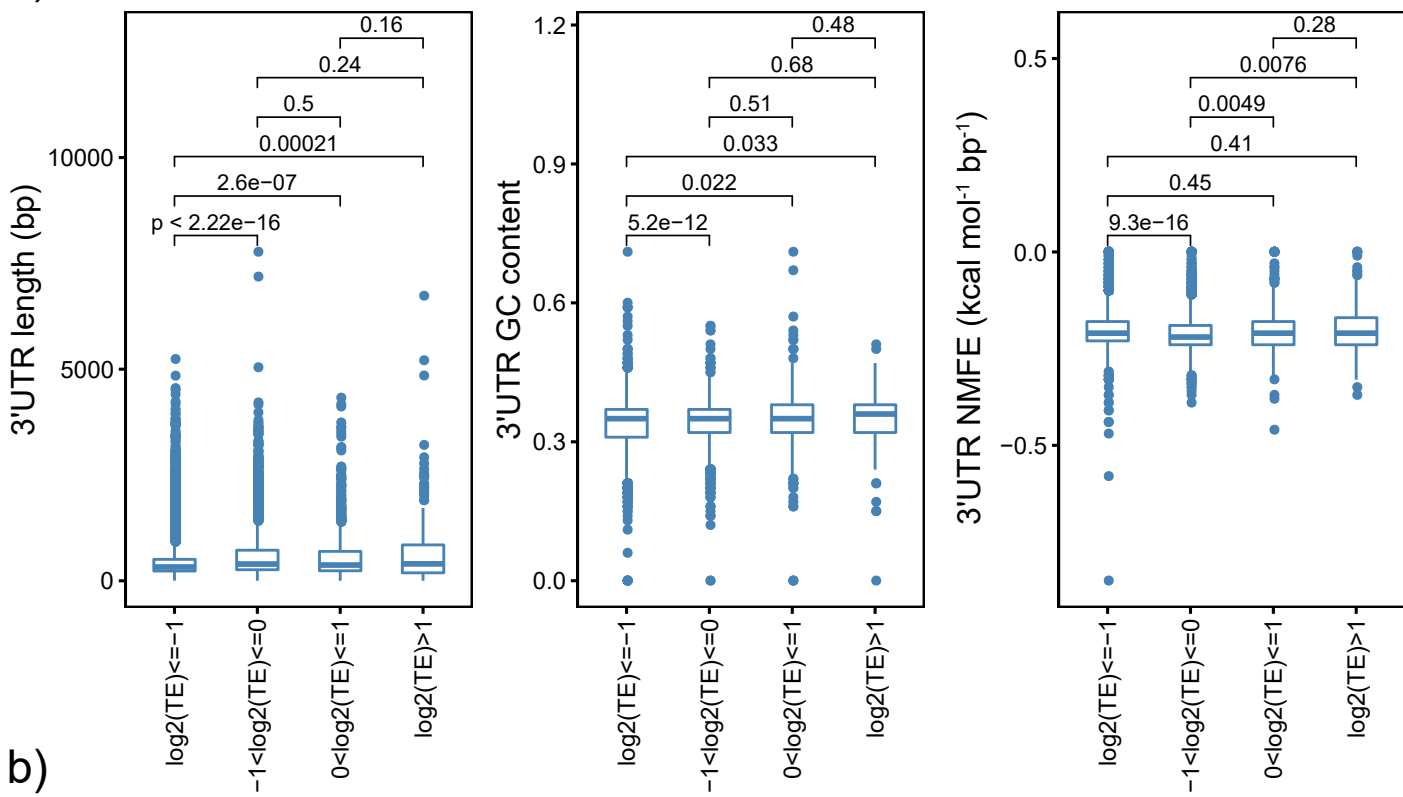

b)

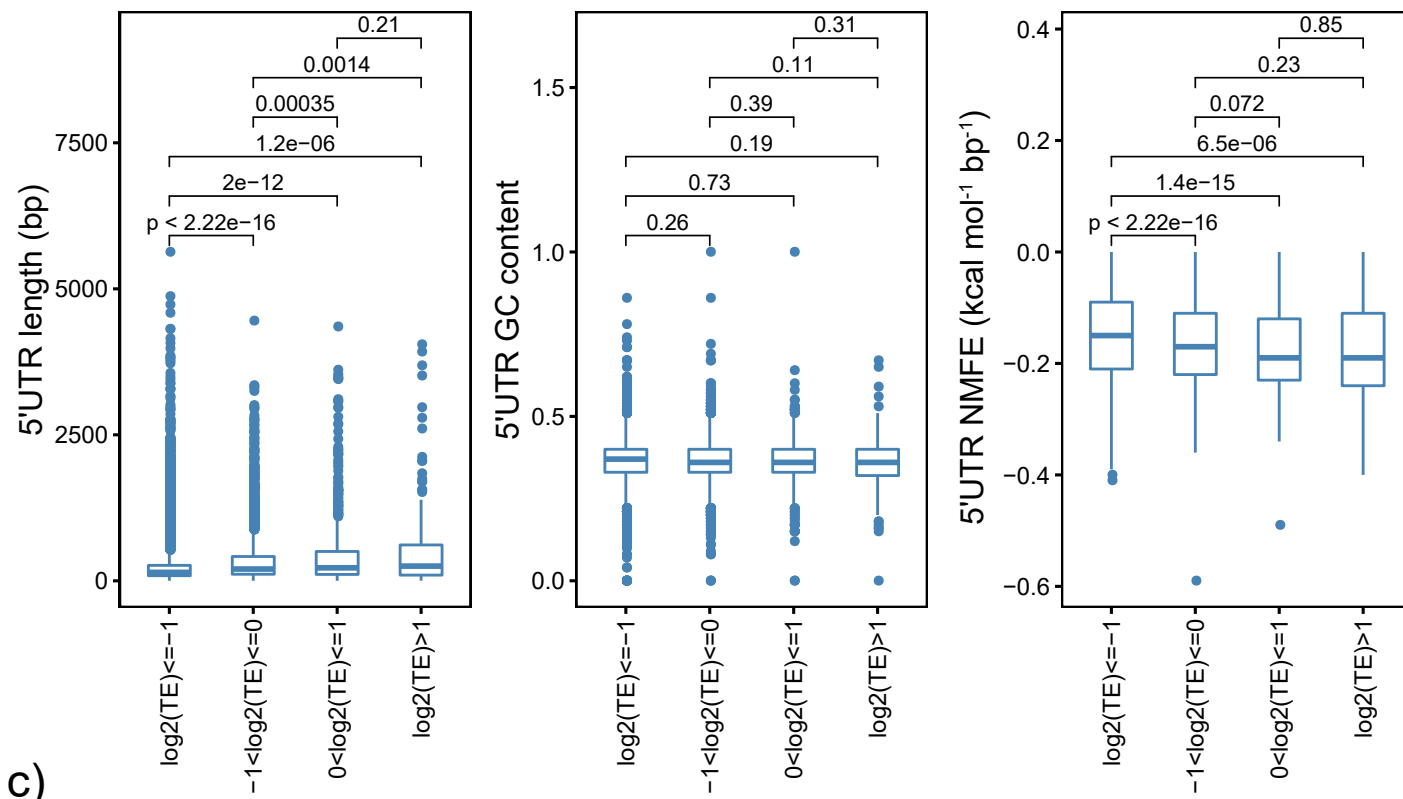

c)

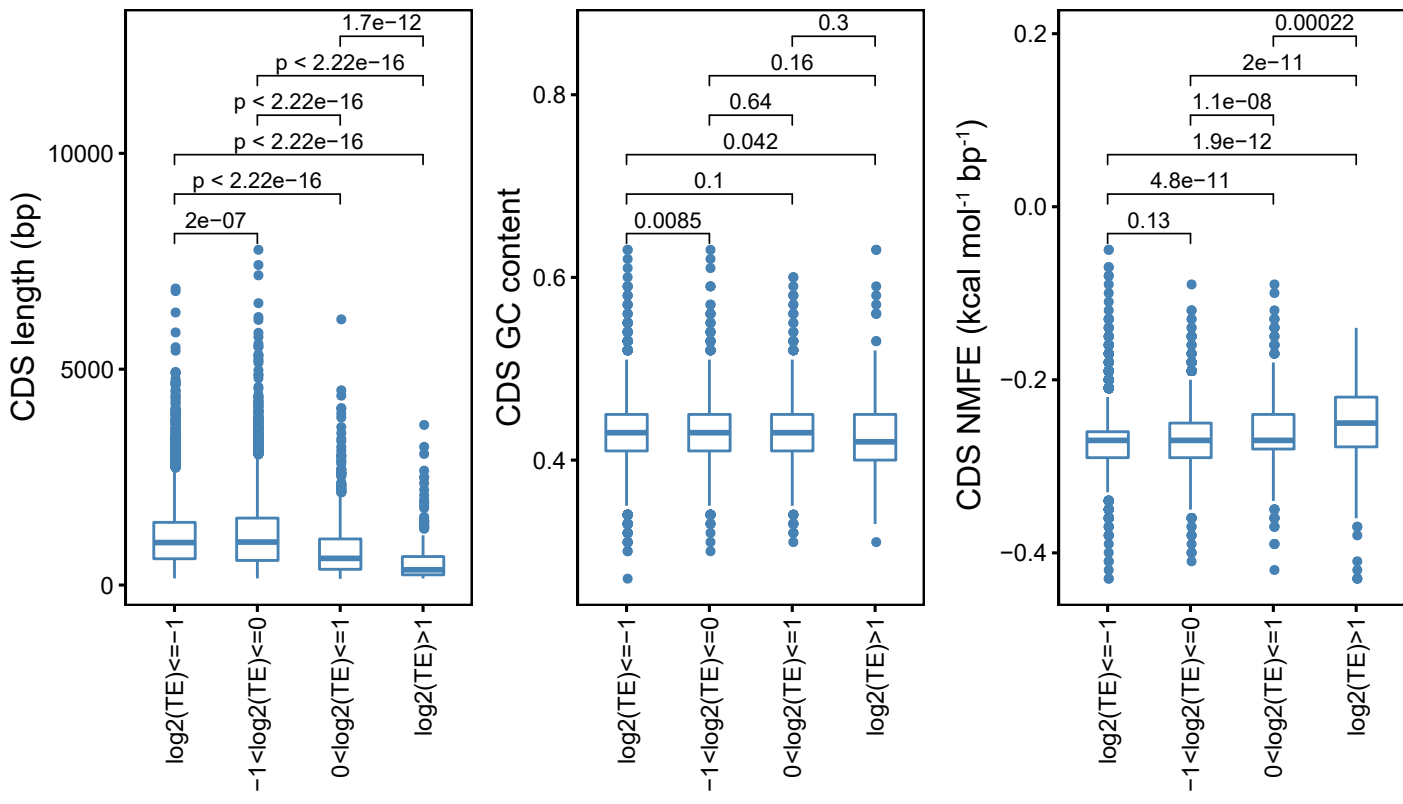

Supplement: Supplementary file 1 [file plants-12-02232-s001.zip › Supplementary files/Fig. S4.pdf]

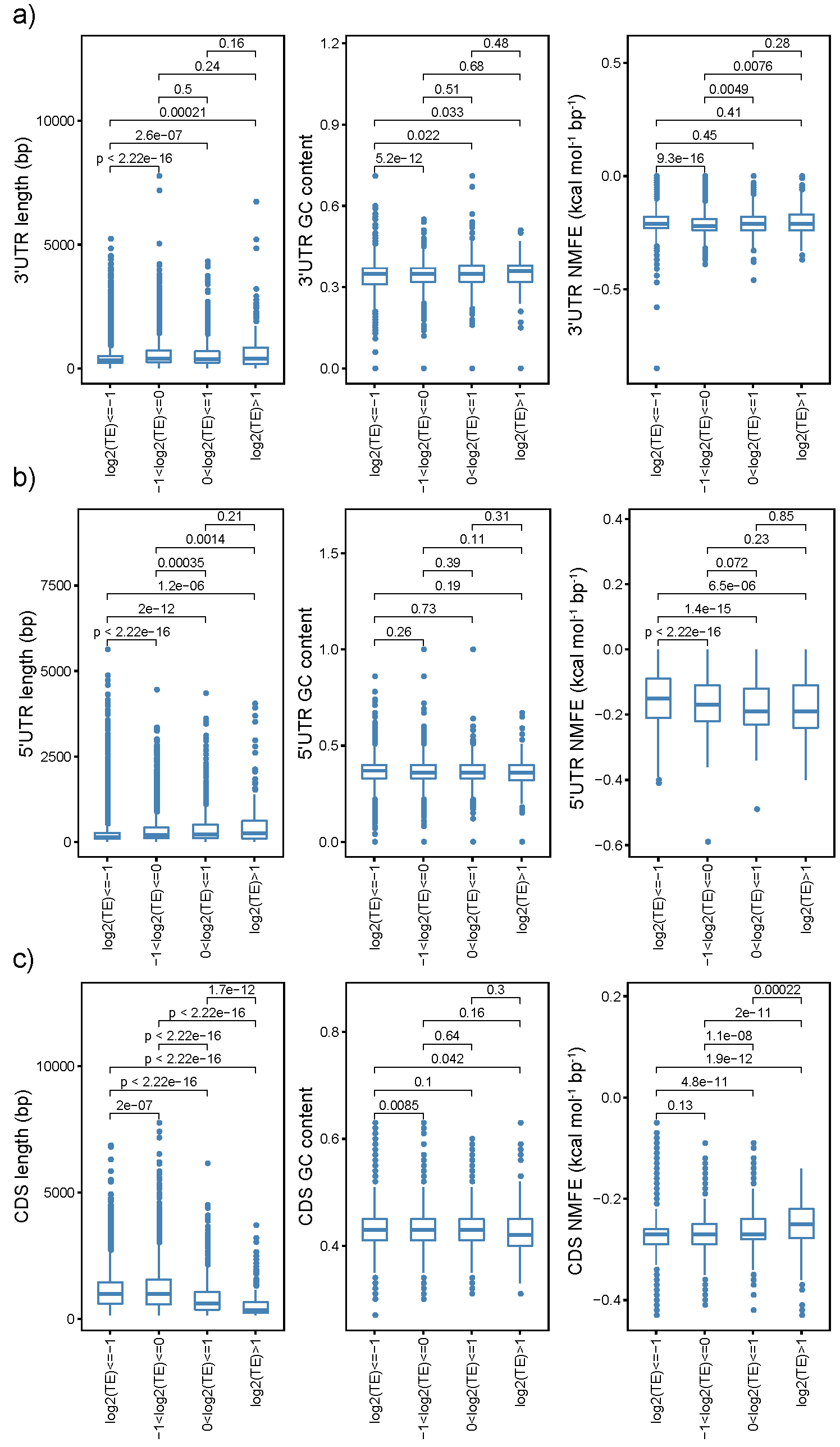

Supplement: Supplementary file 1 [file plants-12-02232-s001.zip › Supplementary files/Fig. S4.tif]

a)

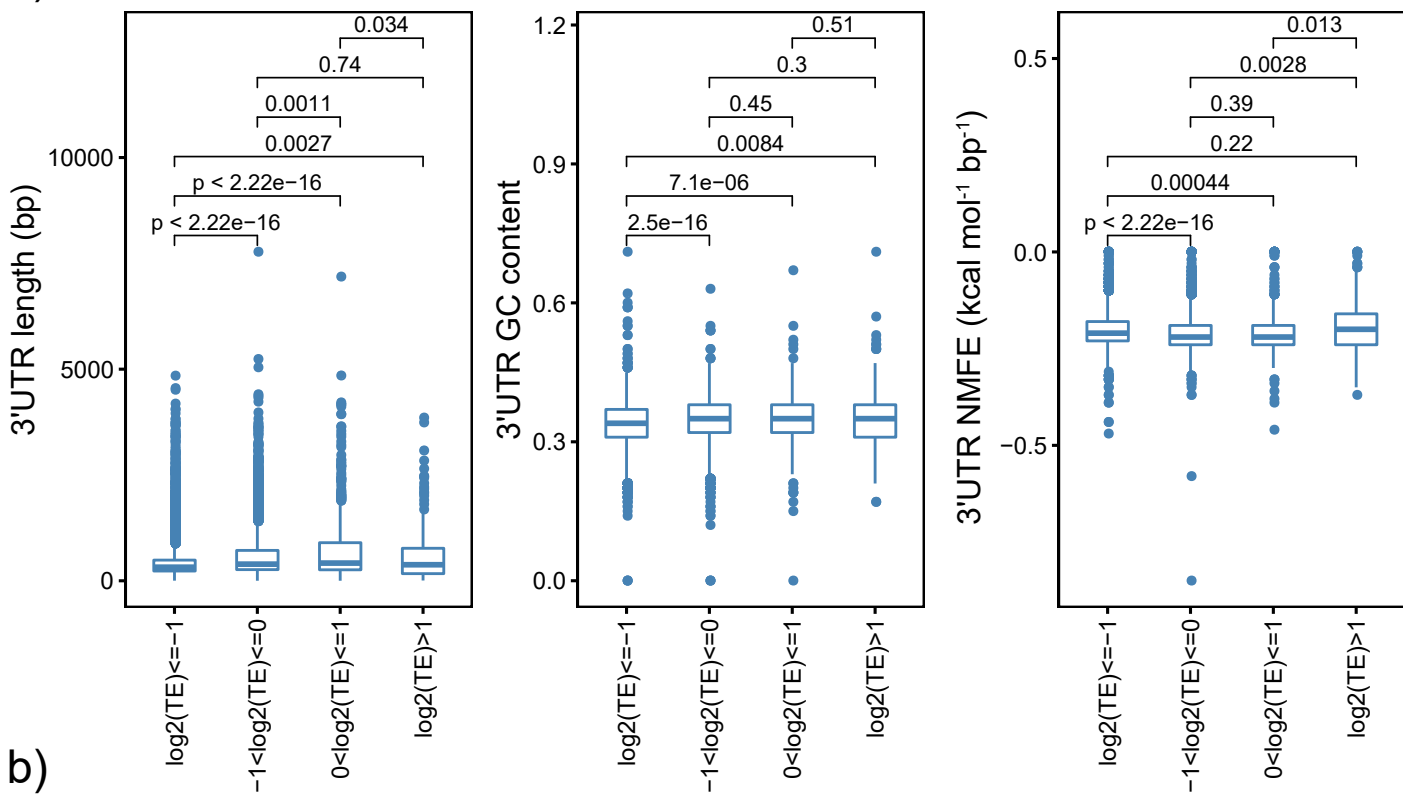

b)

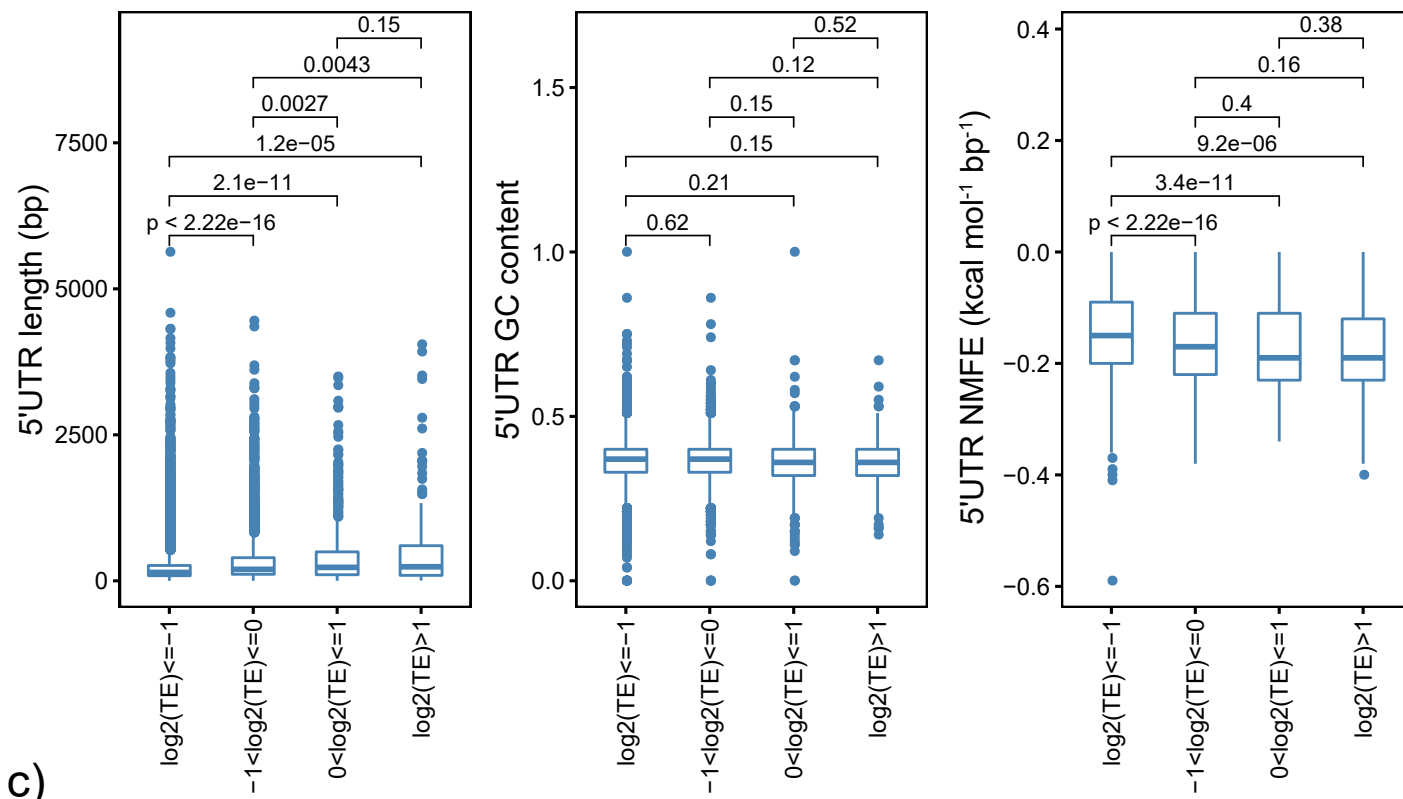

c)

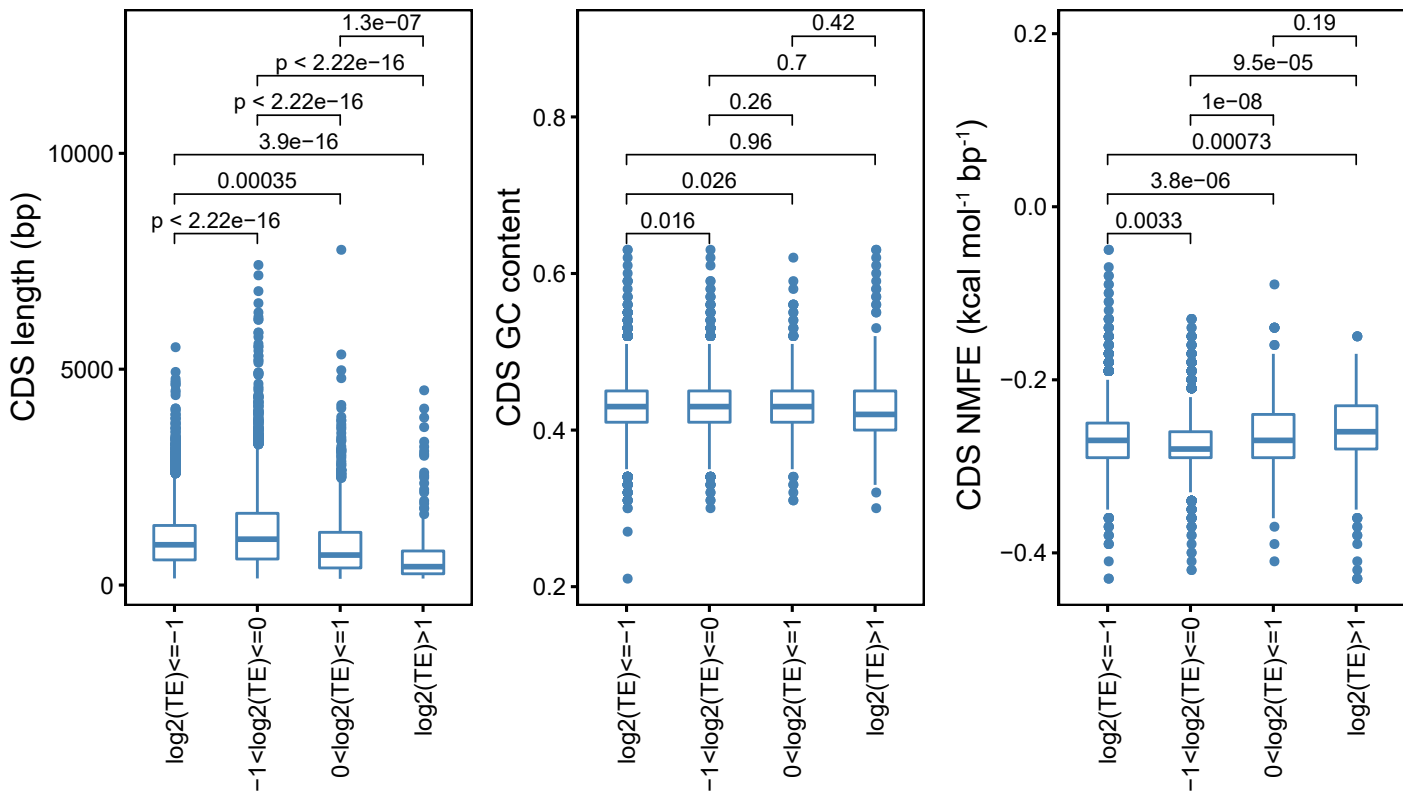

Supplement: Supplementary file 1 [file plants-12-02232-s001.zip › Supplementary files/Fig. S5.pdf]

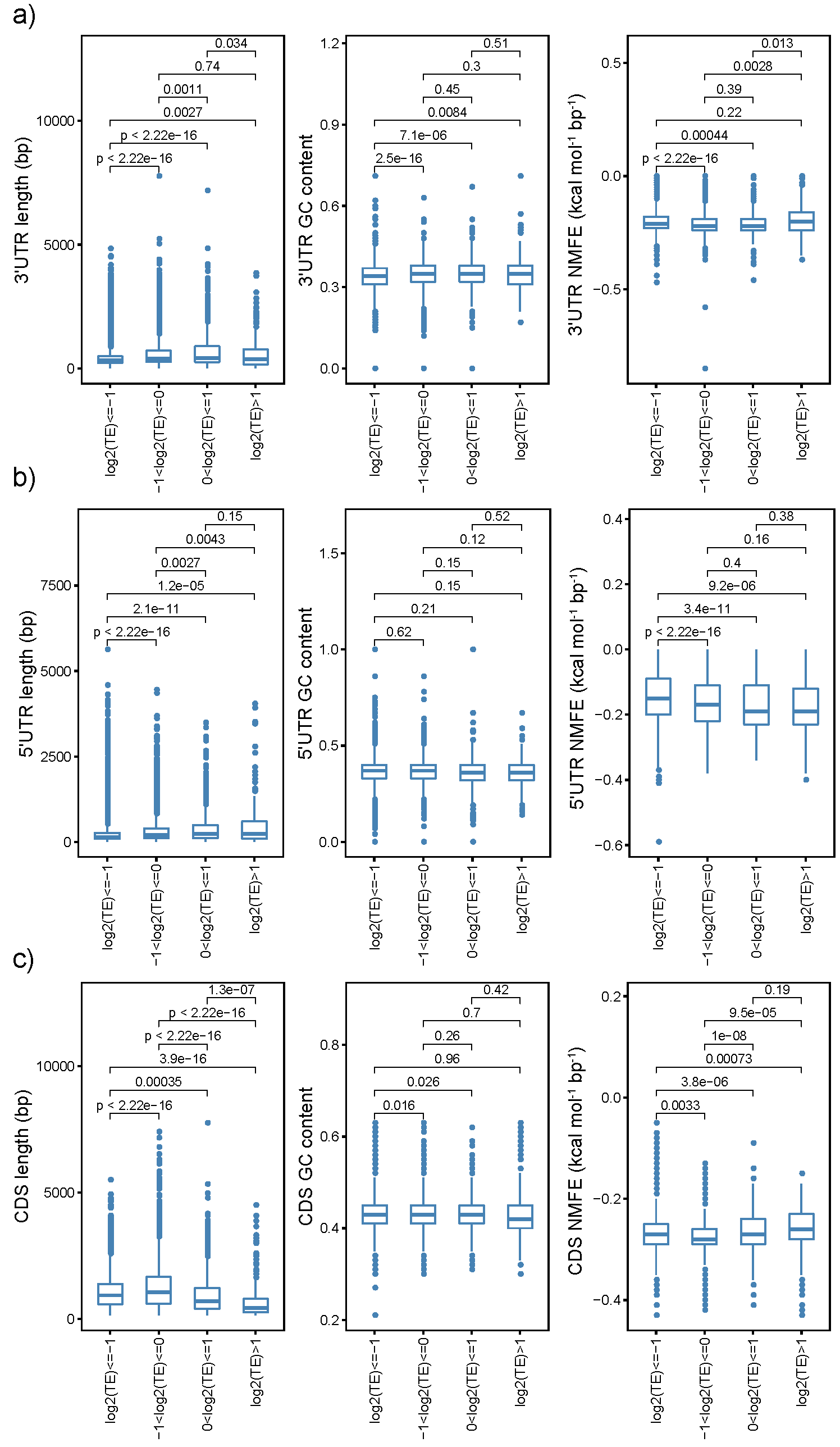

Supplement: Supplementary file 1 [file plants-12-02232-s001.zip › Supplementary files/Fig. S5.tif]

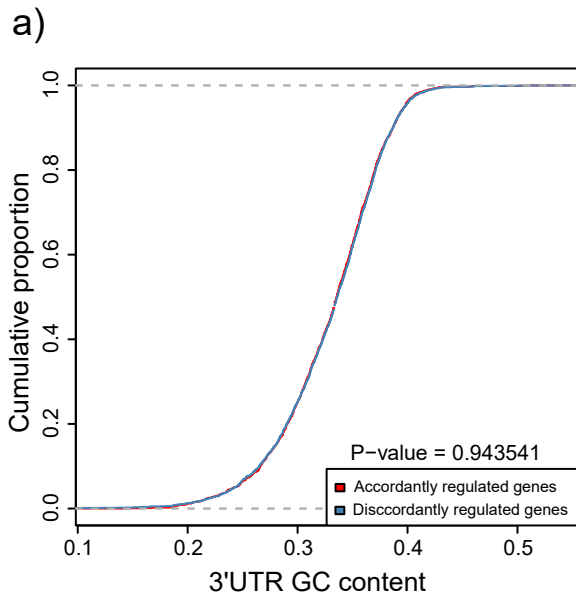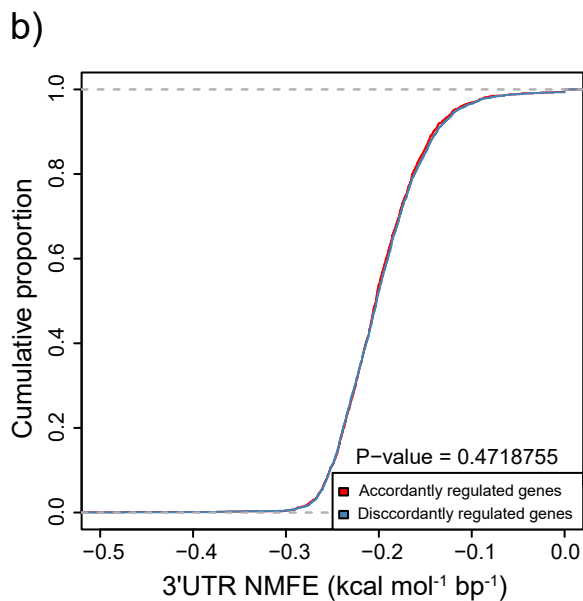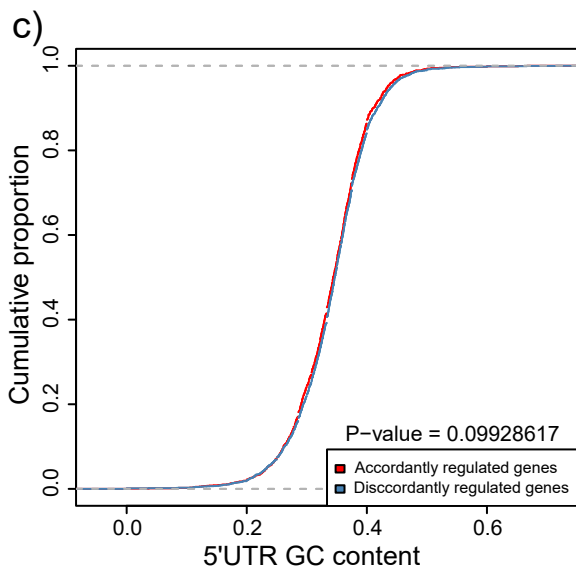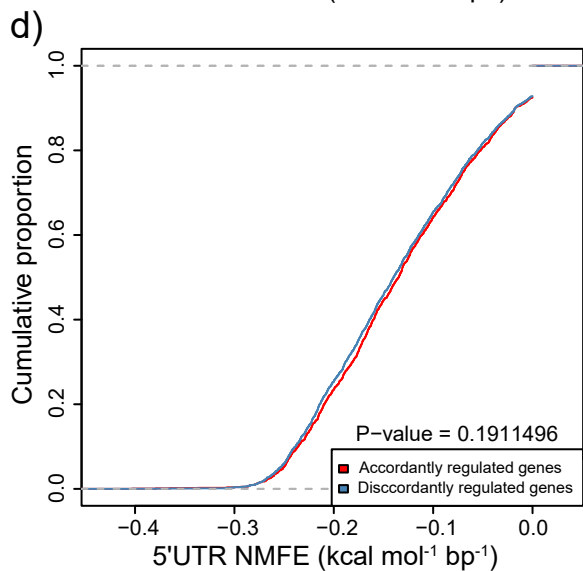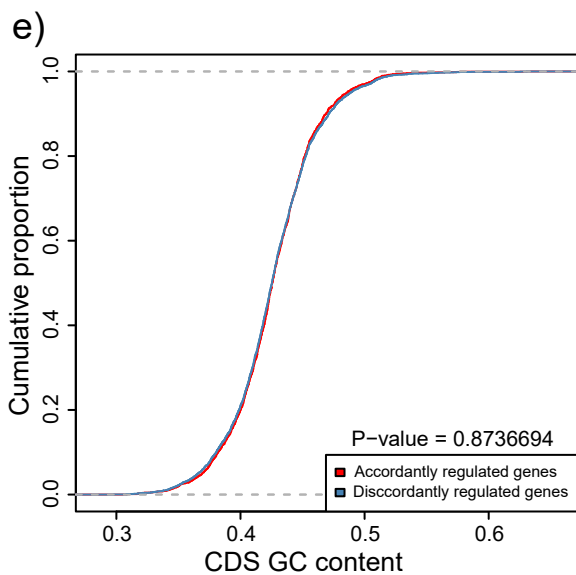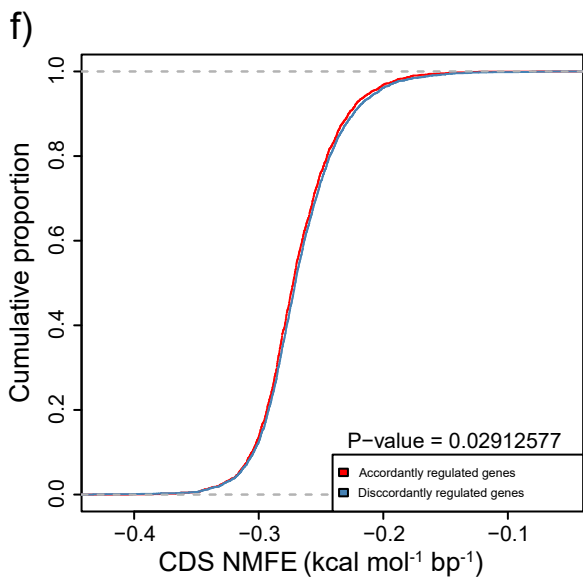

Supplement: Supplementary file 1 [file plants-12-02232-s001.zip › Supplementary files/Fig. S6.pdf]

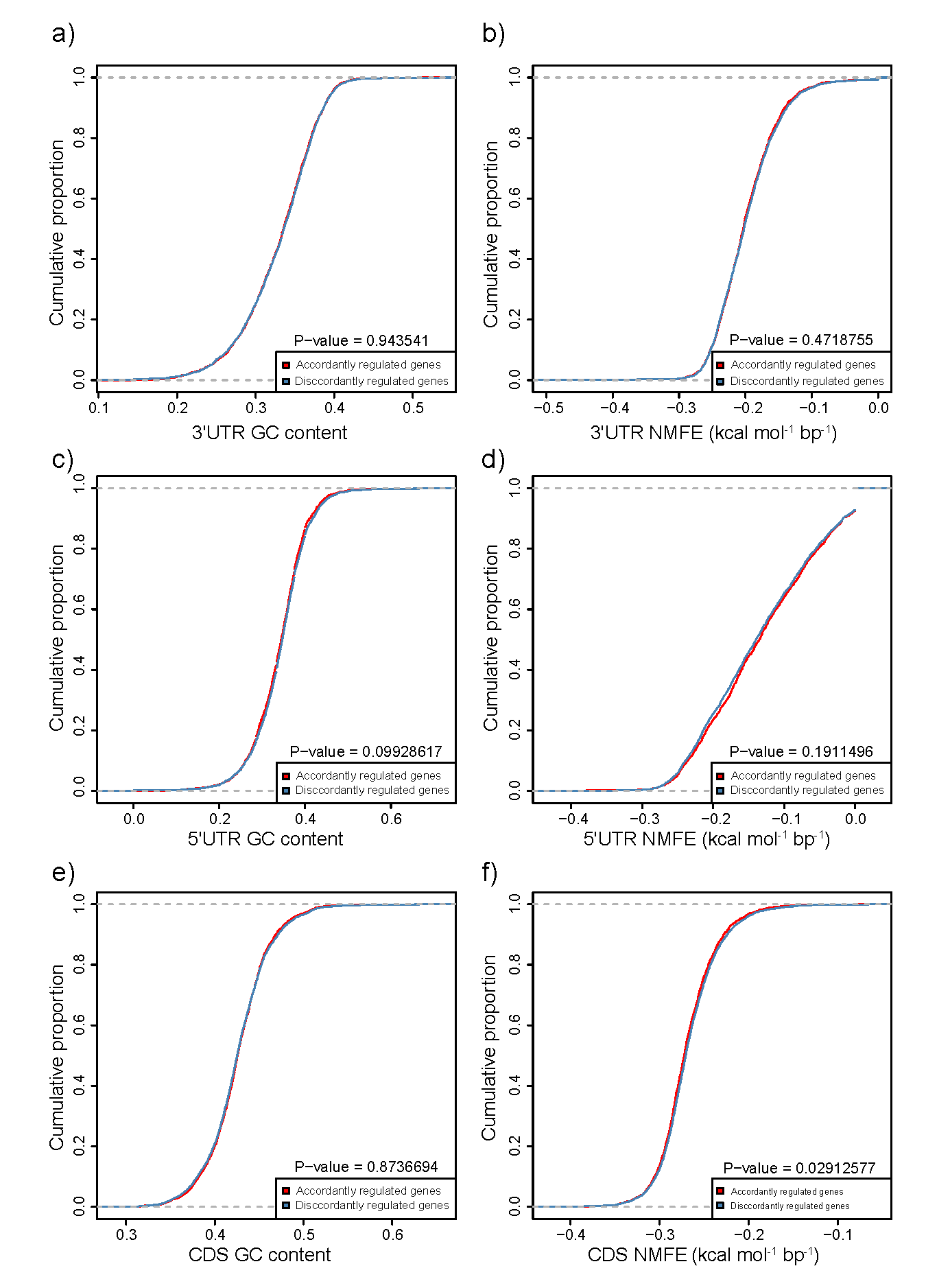

Supplement: Supplementary file 1 [file plants-12-02232-s001.zip › Supplementary files/Fig. S6.tif]

a)

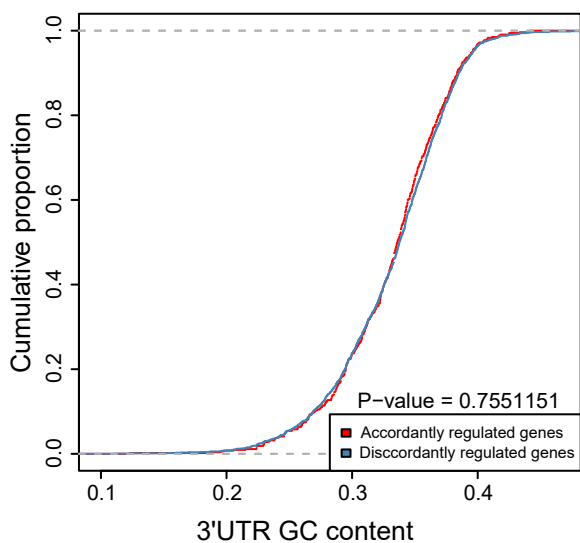

b)

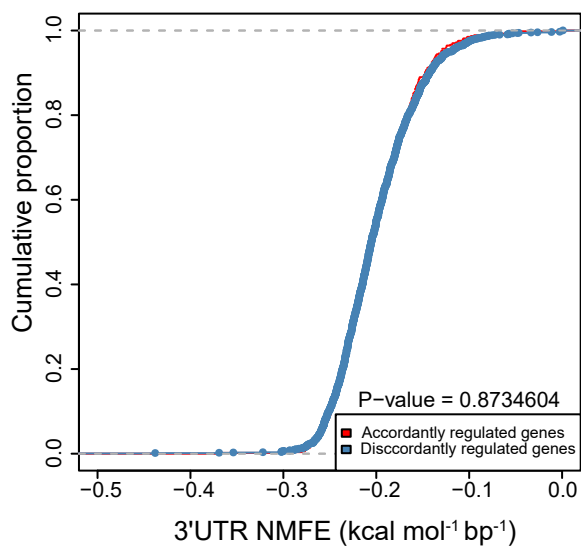

c)

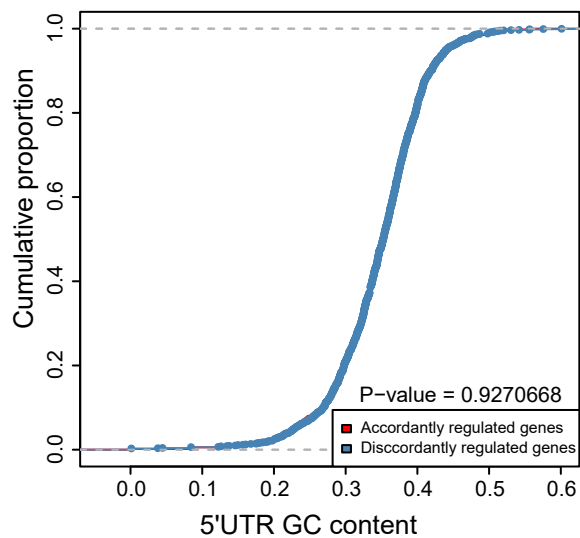

d)

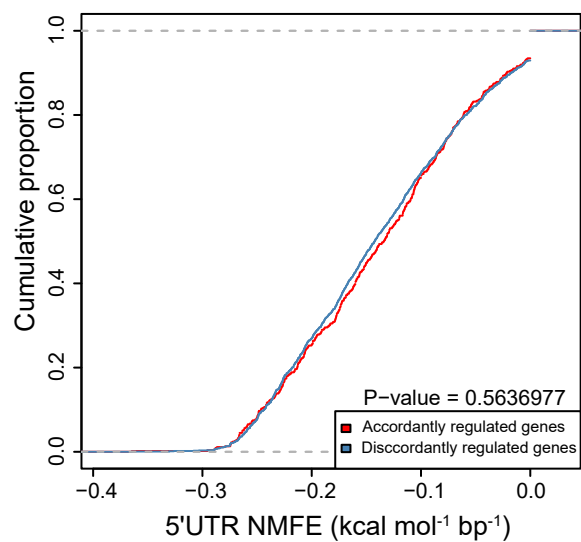

e)

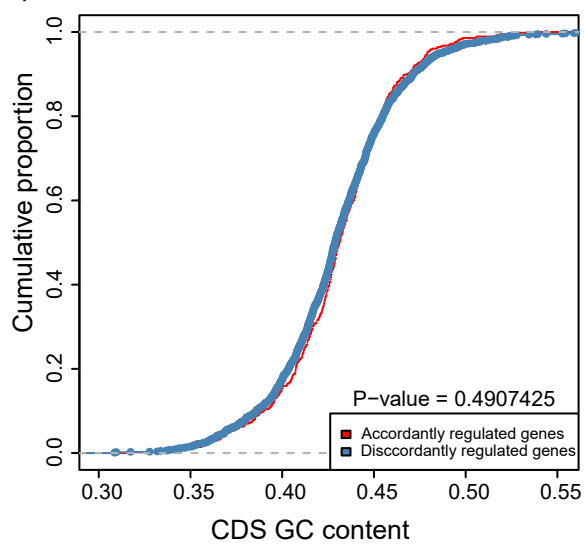

f)

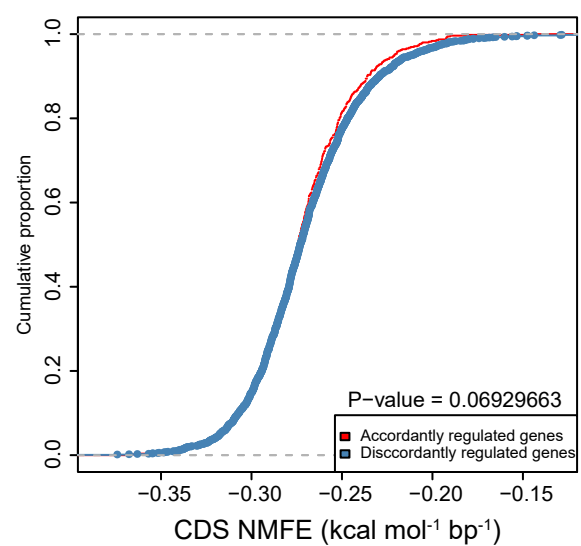

Supplement: Supplementary file 1 [file plants-12-02232-s001.zip › Supplementary files/Fig. S7.pdf]

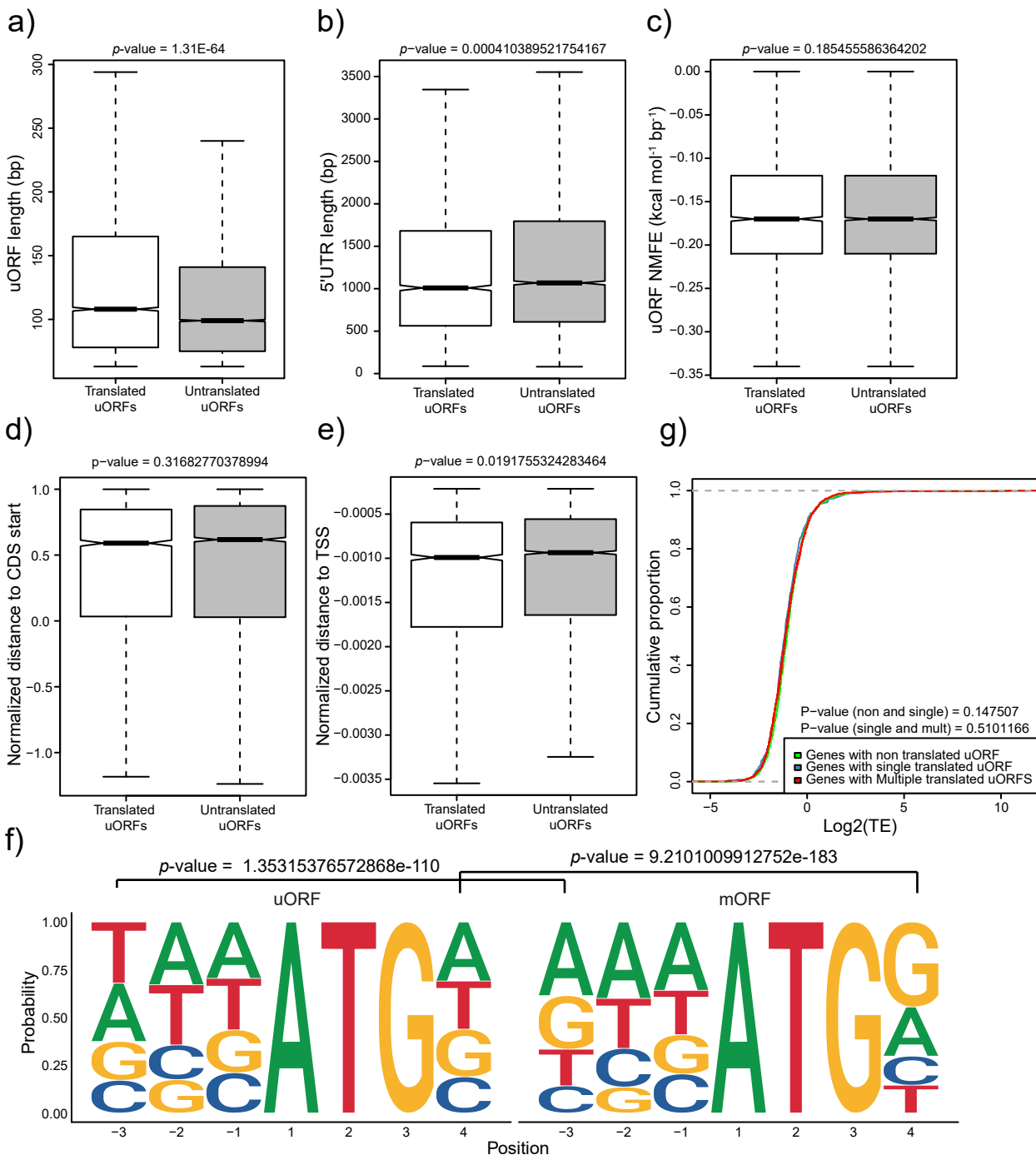

Supplement: Supplementary file 1 [file plants-12-02232-s001.zip › Supplementary files/Fig. S8.pdf]

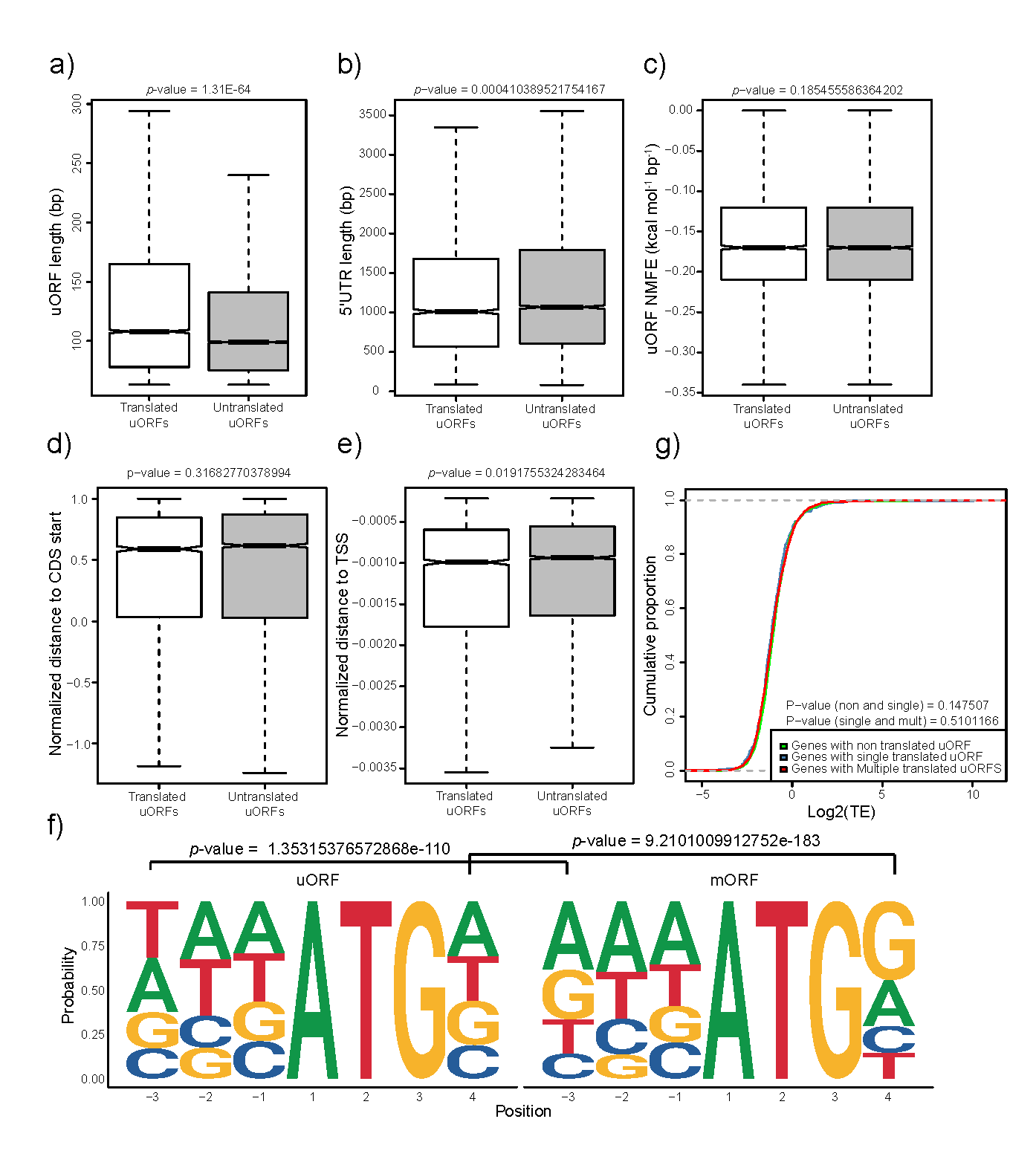

Supplement: Supplementary file 1 [file plants-12-02232-s001.zip › Supplementary files/Fig. S8.tif]

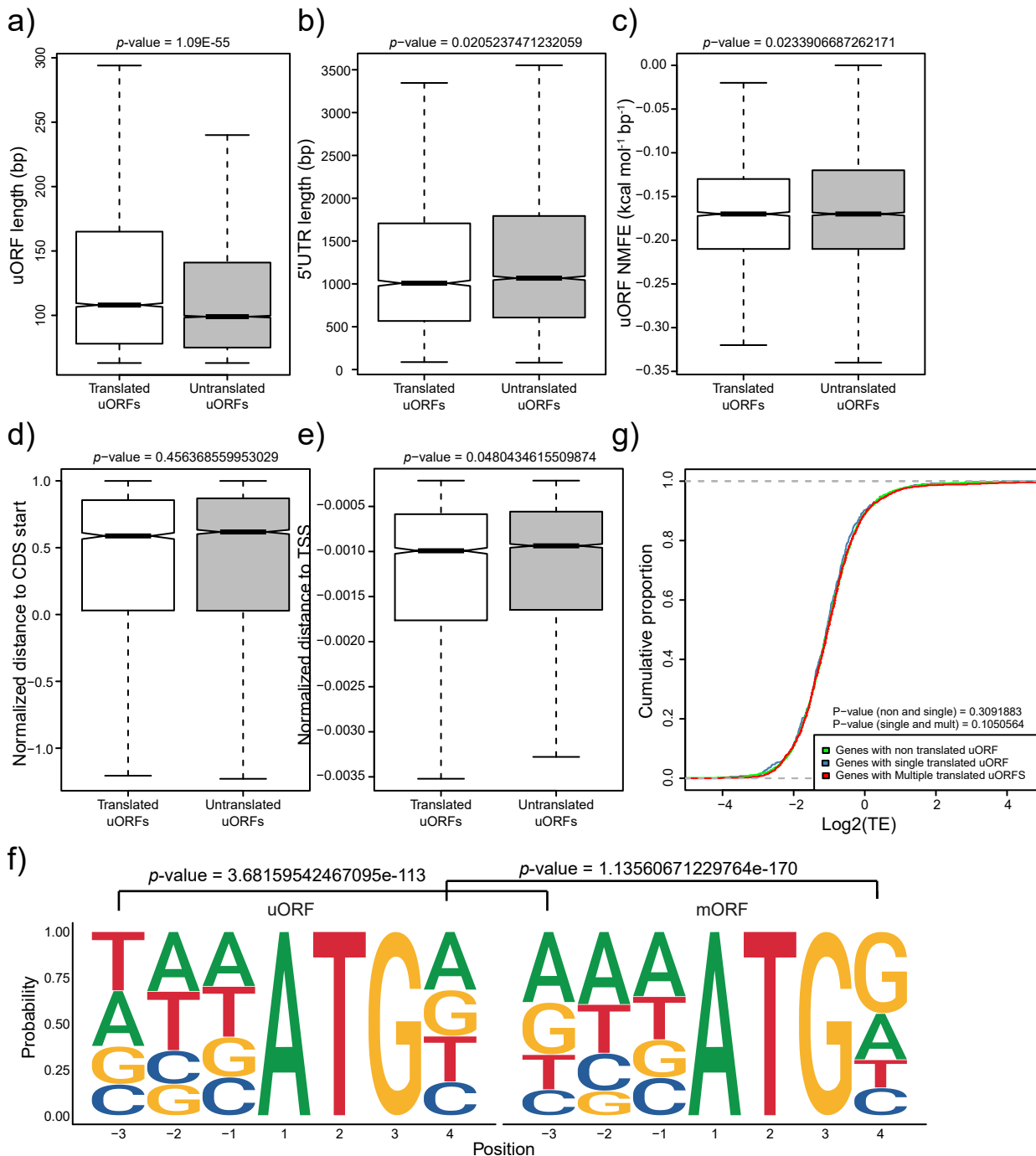

Supplement: Supplementary file 1 [file plants-12-02232-s001.zip › Supplementary files/Fig. S9.pdf]

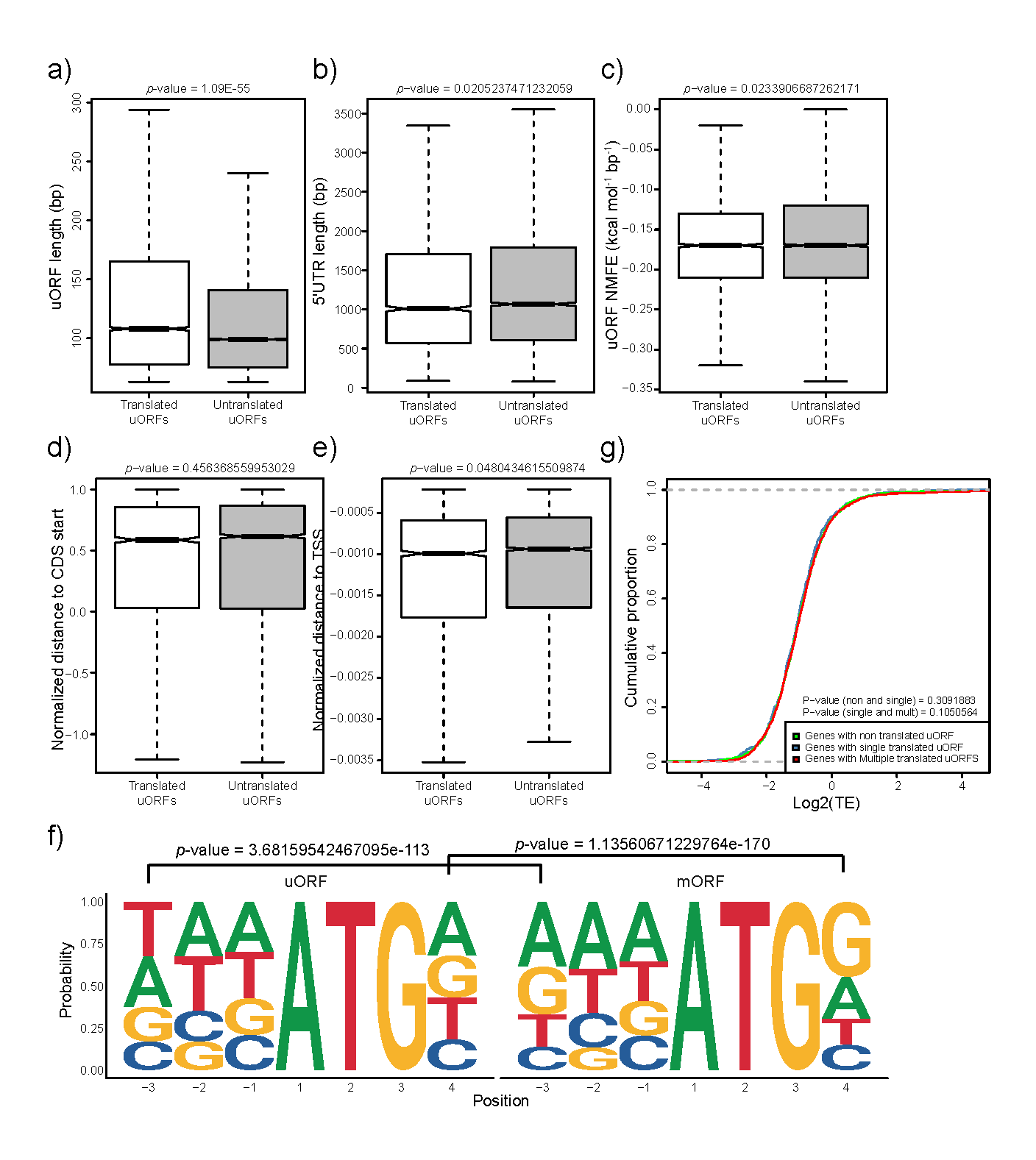

Supplement: Supplementary file 1 [file plants-12-02232-s001.zip › Supplementary files/Fig. S9.tif]
